# Are Expert Patients an Untapped Resource for ART Provision in Sub-Saharan Africa?

**DOI:** 10.1155/2012/749718

**Published:** 2012-04-19

**Authors:** Tom Decroo, Wim Van Damme, Guy Kegels, Daniel Remartinez, Freya Rasschaert

**Affiliations:** ^1^Médecins Sans Frontières, Avenue Eduardo Mondlane 38, Tete, Mozambique; ^2^Department of Public Health, Institute of Tropical Medicine, Antwerp, Nationalestraat 155, 2000 Antwerpen, Belgium; ^3^Médecins Sans Frontières, Maputo, Mozambique

## Abstract

Since the introduction of antiretroviral treatment, HIV/AIDS can be framed as a chronic lifelong condition, requiring lifelong adherence to medication. Reinforcement of self-management through information, acquisition of problem solving skills, motivation, and peer support is expected to allow PLWHA to become involved as expert patients in the care management and to decrease the dependency on scarce skilled medical staff. We developed a conceptual framework to analyse how PLWHA can become expert patients and performed a literature review on involvement of PLWHA as expert patients in ART provision in Sub-Saharan Africa. This paper revealed two published examples: one on trained PLWHA in Kenya and another on self-formed peer groups in Mozambique. Both programs fit the concept of the expert patient and describe how community-embedded ART programs can be effective and improve the accessibility and affordability of ART. Using their day-to-day experience of living with HIV, expert patients are able to provide better fitting solutions to practical and psychosocial barriers to adherence. There is a need for careful design of models in which expert patients are involved in essential care functions, capacitated, and empowered to manage their condition and support fellow peers, as an untapped resource to control HIV/AIDS.

## 1. Introduction

By the end of 2010, approximately 5,1 million people were receiving antiretroviral therapy (ART) in sub-Saharan Africa (SSA) representing only 49% of people in need of treatment [1]. For those fortunate enough to access ART, HIV infection became a chronic disease requiring lifelong treatment. However, retention in ART is a huge challenge. In 2010, a systematic review reported 80.2% (CI 78.0–82.4%), 76.1% (CI 72.4–79.7%), and 72.3% (67.4–76.9%) retention in ART at 12, 24, and 36 months of treatment, respectively, [2].

Frequently mentioned barriers to adherence in SSA are stigma, forgetfulness, lack of awareness, and loss of employment due to frequent absences from work. Shortage of human resources results in inaccessible staff, long waiting times, and poor quality of health services. Moreover, long distances to health facilities, scarce availability and high cost of transport limit access [3, 4].

Although barriers to retention in ART are well studied, they remain in place as a large number of patients keep on defaulting. We postulate that the dominant provider-centred ART delivery model needs to be challenged, taking into account supporting factors for adherence such as adequate information, sense of self-worth, acquisition of problem solving skills, community support, and self-management [4–7].

When PLWHA use their insider's knowledge about their condition, develop self-management skills, have worked out what services exist and when and how to access them, and make day-to-day decisions to enhance their well-being, they can be considered expert patients, exerting a significant control over their own lives. This perception can be accelerated by involving actively PLWHA as expert patients in medical ART care [8].

ART care is a combination of several types of functions such as complicated clinical decision making, routine clinical checkups, ART provision, adherence counselling, social support, and laboratory monitoring. Some of these functions can be standardized and simplified to involve less qualified health cadres and PLWHA in ART delivery [9]. Various care models showed the added value of involving PLWHA in psychosocial and adherence support [5, 10, 11]. However, we believe that more functions, including standardized medical functions such as ART provision could be shifted towards PLWHA to obtain a more demedicalized ART delivery model, primarily based on the communities and expert patients, with professional backup when needed [8, 9]. This participation of PLWHA as expert patients in standardized medical tasks is proposed to streamline the process to access ART, to improve adherent behaviour, psychosocial wellbeing and retention in care [6, 8, 9, 12]. The result is a modified patient-professional partnership, defined by the paradigm of collaborative care. Both patients and healthcare workers can use their expertise, patients as experts about their lives and needs and healthcare workers as experts about diseases [13].

For the scope of this paper, we analysed how PLWHA can become expert patients and get involved in ART provision. To do so, we developed a conceptual framework in which self-management can reinforce PLWHA to become expert patients. We reviewed the literature for examples of involvement of PLWHA in ART provision. We focused on the context of SSA, where staggering workforce shortages limit the response to the HIV epidemic, and where social support networks are an essential element of personhood [14].

## 2. Methods

**Figure 1:**
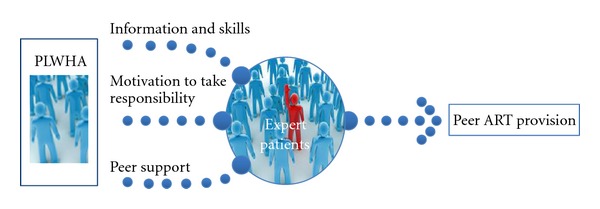
Conceptual framework: elements required to reinforce PLWHA to become expert patients.

Based on our own experience, working in an HIV program in Tete, Mozambique, where patients are involved in the ART provision through community ART groups, we wanted to analyse how PLWHA can become expert patients and get involved in ART provision. In order to structure our thinking about the involvement of PLWHA as expert patients, we developed a framework to illustrate the different elements required to reinforce HIV self-management and to empower PLWHA to become engaged in the care for their condition as expert patients (Figure 1).

To increase their level of self-management and to become expert patients, PLWHA need to be (1) adequately informed and capable of acquiring self-management skills, (2) motivated to take day to day responsibility for their own care, and (3) part of a peer support network [6].

In order to compare our own experience with other HIV care delivery models featuring involvement of PLWHA in ART provision in SSA, we searched the literature on PubMed, the Cochrane database, and Google Scholar from 2000 to 2011. In addition, we reviewed article reference lists and searched websites of WHO and international HIV agencies. A combination of key search terms “SSA”, “HIV”, “AIDS”, “delivery of health care”, “community networks”, “community health services”, “peer support”, “self-help groups”, “expert patient”, “self-management”, “treatment outcomes,” and “retention in care” was used. The search was limited to English literature. All retrieved abstracts were reviewed by FR and TD. We only selected articles that explicitly described PLWHA involvement in ART provision in SSA.

Papers on psychosocial and adherence support and ART provision by lay providers, who were not explicitly PLWHA, were excluded, as the added value of these strategies is already extensively described in the literature. Also studies with PLWHA involvement during a short time period were not considered as we focused on long-term solutions to empower PLWHA.

We applied the conceptual framework on the selected studies.

## 3. Results

The literature search for involvement of PLWHA as expert patients in ART provision in SSA produced 50 articles. Twenty-four articles were excluded as they did not reveal information on PLWHA involvement in care or ART provision. Of the 26 remaining articles, 6 were excluded as they described a nurse-based care model without an explicit involvement of PLWHA. Another 9 articles were excluded as lay providers were involved in psychosocial support and adherence support, but not in ART provision, and PLWHA were not involved or PLWHA involvement was not explicitly described.

Three articles illustrated the positive impact of involvement of PLWHA in psychosocial and adherence support on adherence and treatment outcomes. But as these articles did not mention an involvement of PLWHA in ART provision, they were excluded for further analyses [5, 10, 11].

In addition, we excluded 4 articles from rural Uganda where community health care workers (CHWs) or volunteers provided ART at patients' homes without involving PLWHA. The CHWs were trained, salaried, and equipped with a motorbike and mobile phone, whereas social recognition was the main motivation for the volunteers. Outcomes obtained were comparable with conventional care. It is hypothesized that these ART delivery models, primarily based on CHW and volunteers, could serve as a role model for further involvement of PLWHA in ART provision and care [15–18].

**Figure 2:**
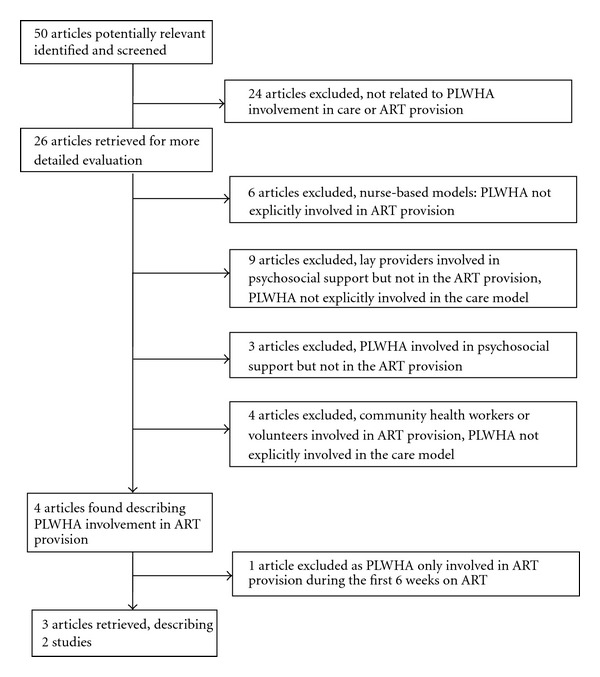
Results of literature search on PLWHA involvement in ART provision.

We only found four articles, including our own experience in Tete, involving PLWHA in ART provision in SSA. One article described the increased medication adherence as a result of a peer-delivered direct observed treatment (DOT) strategy during the first 6 treatment weeks of antiretroviral treatment at a health facility in Mozambique [19]. This study was further discarded, as PLWHA were involved only in the initial 6 weeks of ART provision, and since to our understanding DOT is not compatible with the proposed process of reinforced self-management for PLWHA to become expert patients.  Figure 2 describes the research strategy and results.

Table 1 summarizes the three articles retrieved describing PLWHA involvement in ART provision. We did not find published examples of PLWHA involved in decision making of ART initiation.

The first two articles discuss the same cluster randomized controlled trial in *Kenya*, comparing a community-based care model to a conventional-clinic-based care model. Trained PLWHA, known as Community Care Coordinators (CCC), delivered ART monthly at the patients' homes and referred patients if clinical problems occurred. Every three months patients were invited for a routine visit at the clinic. Each CCC took care of 8 to 20 clinically stable adult patients on ART. Patients perceived CCC as their confidents and advocates. By playing this role, CCC obtained insights into adherence and psychosocial issues. The clinic regarded the CCC as an extension of the clinic staff. The outcomes obtained were comparable to those with conventional care but clinic visits were reduced by 50%. The authors concluded that trained PLWHA have the additional advantage of their day to day experience of living with HIV [20, 21].

We documented our own experience in *Mozambique*, where PLWHA are involved in community ART provision with as main objective to improve the retention in care and relieve the health facilities. Adult patients stable on ART were invited to self-form peer groups of maximum six members, called community ART groups (CAG). Interested patients were capacitated during an information session on the CAG dynamic, including community ART delivery, adherence support, and social support. Monthly the CAG members meet in the community to check pill counts, to verify the health status of members, and to choose a representative to travel to the clinic. He/she reports the adherence and health outcomes and collects a drug refill for all CAG members at the clinic. Every six months, all members are invited for a routine clinic visit, CD4 check and an interactive group session. This dynamic is driven by mutual support and the need to obtain an easier refill, integrated in the patients' daily life in the community. No financial or material incentives were given. Of the patients in CAG, 97.5% were retained after a median follow-up time of 12.9 months [22].

Both programs fit in the concept of the expert patient, as PLWHA were motivated to acquire skills to manage their condition and to support fellow peers, using their day-to-day experience of living with HIV (Table 2). In both programs PLWHA functioned as partner in care and a bidirectional referral system was installed, as sick patients were referred to the clinic and the community network was used for tracking of patients lost to followup.

In both Kenya and Mozambique PLWHA acquired skills to provide ART, to refer sick patients to the clinic, to report on treatment outcomes, and to give psychosocial support [20–22]. In Kenya, CCC were trained in medical and psychosocial tasks to become expert patients. Being PLWHA, they were able to understand and resolve psychosocial barriers to adherence of their peers in the community to an extent that was not obtained during consultations at the health facility [20, 21]. In Mozambique, all patients in CAG were engaged on a voluntary basis and capacitated to participate in the daily care of themselves and their peers. PLWHA self-formed support groups and met monthly in the community to share information and problem-solving skills regarding physical and psychosocial issues. Major problems were reported to the health staff and feedback was debated at the next meeting in the community, resulting in an information loop between health facilities and the community [22].

In Kenya the CCC benefitted from a formal training and were remunerated as CHW. There was a solid monitoring system in place to facilitate regular control and supervision by the healthcare workers, and CCC were held accountable for their daily performances [20, 21]. In Mozambique the peers were involved from the start in the process of planning and implementation of the CAG dynamic, which resulted in a feeling of ownership and motivated the PLWHA to stay involved in the CAG dynamic. Affordability and accessibility of ART refill was improved for CAG members. These direct benefits were a straightforward incentive to motivate PLWHA to become engaged in their own care [22].

In both countries the motivation for expert patients to take responsibility was strengthened through the proximity with their fellow peers in the community, as they added their day-to-day expertise of living with HIV to their acquired knowledge. This resulted in unique relationships among peers, built on common needs, confidence, and reciprocity [20–22]. CCC in Kenya were perceived by the patients as their advocates, a relationship which enabled them to bring practical solutions for psychosocial problems related to adherence where the traditional care providers had failed, and to ensure the communication between the PLWHA in the community and the healthcare workers [20, 21].

## 4. Discussion

### 4.1. Expert Patient in Chronic Lifelong Conditions

The increase in chronic lifelong conditions (CLLCs), such as diabetes and hypertension, is severely burdening health systems in many countries. The commonly used provider-centred models are resource intensive, as they require high inputs in skilled health workers. Consequently, it is becoming increasingly difficult to keep up with growing needs and demands of people living with CLLC, even in high-income countries.

Therefore, there is a growing interest in strengthening self-management, putting patients with CLLC at the centre of a web of support, surrounded by expert patients, peer groups, and health professionals. In this way, patients could be empowered to manage their own CLLC and take full responsibility for their conditions and their lives [23]. This is thought to have the potential not only to contain the escalation of health care costs, but also to improve outcomes. People for whom a CLLC is diagnosed reach a turning point in their life. They need to “rebalance” their daily habits to the requirements of their condition and play as such a crucial role in the management of their condition.

Indeed, people living with CLLC take daily decisions linked to pill intake and interpretation of signs and symptoms, requiring often subtle adaptations in diet or behaviour. Such daily adaptations and long-term management often turn them into real experts in living with a chronic condition. Their engagement can be expanded and patients can become more autonomous, as already commonly practiced in diabetes care in high-income countries.

We believe that there is a need to frame HIV/AIDS as a CLLC, requiring lifelong adherence to medication. When PLWHA acquire self-management skills and are empowered to become expert patients, their physical and psychosocial well-being is expected to improve as PLWHA will function more autonomously, which is essential to sustain lifelong adherence to treatment. In addition, their dependency on scarce skilled medical staff is expected to decrease. The latter is particularly relevant for SSA, where the burden for HIV is the highest and health systems the weakest, and where big proportions of populations remain unattended to today [8, 9, 12]. Involvement of PLWHA as expert patients in simplified care tasks, which represent the bulk of HIV services, can relieve the weak health systems in most SSA countries with high HIV prevalence, in order to cope with the increasing patient load. Hence, involvement of PLWHA as expert patients can bring advantages for both the provider and the patient.

A Cochrane review showed that adequate information and practice of medication management skills among PLWHA resulted in better adherence, which is known to result in better treatment outcomes [7]. The process of information exchange can be boosted through social interactions among peers, who add to their acquired knowledge their expertise of day-to-day living with the disease [8, 9]. To reinforce their level of self-management and to become expert patients, PLWHA need to acquire self-management skills for three sets of tasks faced by people with chronic conditions: (1) medical management of their condition such as taking medication and self-monitoring, (2) developing and maintaining supportive social relationships with healthcare workers and community members, and (3) coping with psychological distress such as fear for stigma and anger, frustration, and the sadness of having a lifelong condition (“living positively”) [13, 24].

Adequate information and practice of self-management skills are not enough to achieve and sustain behavioural change. They need to be combined with motivation, defined as the driving force by which humans achieve their goals [6]. The motivation to take responsibility in self-care to achieve adherent behaviour depends on the importance given to treatment and adherence, the confidence in the care provider and the net effectiveness of the treatment, and the self-confidence to be able to manage challenges inherent to lifelong treatment. Group education, shared decision making and social support motivate and empower PLWHA who wish to become better self-managers [6]. Community participation, as described in Mozambique, responding to real needs and involving the communities straight from the start in the planning and the implementation will motivate and empower those engaged and is a precondition for sustainability of activities embedded in the community [25].

To be successful, service delivery in SSA should be adapted to African personhood, grounded in social integrity and the need to maintain social support networks [14]. In our experience with the CAG in Mozambique, social relationship networks among CAG members in a context of economic hardship are used to overcome social, financial, and practical difficulties. They are based on trust, cooperation, reciprocity, and sociability. They contain a silent agreement about being supported and supporting, knowing that these roles can change over time and what is invested will give a return [26]. CAG members in Mozambique have a mutual agreement to support each other on a daily basis. As a result, such social networks can be an important resource to reinforce self-management and adherence [22].

### 4.2. Examples of Expert Patient Involvement in ART Provision in SSA

We looked at the literature for examples of involvement of PLWHA as expert patient in the ART provision in SSA and found only two published examples: one example of trained PLWHA in Kenya and another of self-formed peer groups in Mozambique. In both models, ART refill is dissociated from clinic visits. Routine clinic visits are scheduled three- or six-monthly, respectively, in Kenya and Mozambique. In between, patients can go to the clinic whenever they are sick. Community ART provision improved the accessibility and affordability of ART, as it decreased direct costs for transport and indirect costs due to time spent travelling and queuing in a health facility. Both programs had similar or improved treatment outcomes compared to conventional care and a reduced workload in the health facilities. Both programs fit in the concept of the expert patient, as PLWHA were motivated to acquire self-management skills to manage their condition and to support fellow peers, using their day-to-day experience of living with HIV. The major difference between both programs is that the trained PLWHA in Kenya are remunerated, equipped, and considered as an extension of the health care system, whereas in Mozambique the CAG are more community owned [20–22].

A possible limitation of the literature review is publication bias, selecting only field experiences resulting in positive outcomes. In addition, many interesting experiences are local problem-solving strategies, which are never reported in the scientific literature. However, these pilot models confirm results of recent reviews indicating that task-shifting to PLWHA as expert patients can be effective under certain conditions and can increase significantly the number of services provided and population reached at a given level of quality and cost [27, 28]. There is a need to assess further the potential of care models based on voluntarism in Mozambique versus remunerated expert patients in Kenya in terms of effectiveness, efficiency, and sustainability. For example the cost-effectiveness of both strategies needs to be measured and compared. Additional staff such as peer CHW in Kenya require additional means to pay them salaries and to train and equip them, whereas full responsibility of PLWHA for medical care functions as in Mozambique implies an inevitable loss of control for the provider. It is important to balance both the costs and the yield of each approach, in terms of impact on treatment outcomes and quantity of services provided for a given cost.

### 4.3. Possible Concerns

Often mentioned concerns related to community-embedded ART programs are stigma and fear for reduced quality of care. Stigma is a known barrier to adherence as it threatens social integrity and patients run the risk of becoming isolated. The impact of the visibility of HIV-related activities on stigma in the community needs further investigation. In Kenya peer CHW were named health counsellors to avoid the AIDS label [20]. On the other hand, when PLWHA support each other and form a social network, the negative impact of stigma can be overcome, as documented in Mozambique [29]. In South Africa it was found that buddies, support groups, and CHW predict and sustain treatment success [5].

Quality of care related to task shifting and other innovative models of service delivery is another concern raised. However, when considering survival as the most important indicator of quality of care, peer provision of ART will always compare favourably with no treatment as ART is the only effective clinical intervention to reduce HIV-related mortality [30]. Community-based ART by lay workers in Uganda led to a 90% reduction of mortality among adults and a large reduction of mortality among their children in a population that otherwise would have had difficulties to access care [17].

However, involving PLWHA as expert patients in their own lifelong care is not a panacea. Models engaging communities should not be shaped just to fill the gaps in the health system or to strengthen it. Instead, they should be based on the needs of the PLWHA, thus requiring their involvement in the design and planning of the program [25]. There is a need to develop both the community and the clinical platform as complementary pillars of health systems [31].

### 4.4. Future Prospects for HIV Self-Management

Once the concept of the expert patient is accepted and established, we think there are several further prospects that could be explored. First, once oral HIV tests will be available on African markets, there is a potential to expand testing in communities [32]. Second, the use of point-of-care testing could be further explored. For example, point-of-care CD4 techniques can be integrated in community delivered services, so that trained expert patients could assume medical tasks such as HIV testing, CD4 counts, and possibly deciding on ART initiation for uncomplicated patients [8]. Third, community support and self-management for groups such as adolescents, pregnant women, pre-ART patients and TB patients need to be studied. Fourth, to facilitate access to ART, drug refill points could be integrated in and managed by communities, schools, market places, and the workplace [33]. Fifth, the use of new communication technologies like cell phones and smart phones can contribute to PLWHA empowerment and improved self-management, strengthening communication within the community and with the health sector [34]. And, last but not least, the potential future implementation of a treatment-as-prevention strategy will have to rely heavily on PLWHA' involvement in chronic care and lifelong adherence to make this strategy feasible [35].

## 5. Conclusion

In SSA, a huge proportion of the population needs to adhere daily to life-preserving medication, a process which requires an uninterrupted supply integrated in daily life in the community. We found two innovative pilot models in SSA showing the feasibility of involving PLWHA in tasks such as ART provision. Both models reduced barriers to ART refill, decreased dependency on health services and resulted in health outcomes comparable to facility-based care. Using their day-to-day experience of living with HIV, expert patients were able to provide better fitting solutions to practical and psychosocial barriers to adherence. These results seem consistent with theoretical insights and practical experiences from diabetes and other chronic diseases in the West. An extension of these pilots is needed to evaluate the scalability in different contexts. There will be a need for careful design of such models according to the local contexts and realities to explore if PLWHA can become expert patients, capacitated and empowered to manage their HIV and support fellow peers, as an untapped resource to control HIV/AIDS.

## Figures and Tables

**Table 1:** Study characteristics of included articles.

**Country**	**Inputs**				**Outcomes**	**Method**
	Tasks of PLWHA	Training	Salary	Equipment		
Kenya [20, 21]	*Community care coordinators*, each responsible for 8–20 adults: (i) Monthly drug refills (ii) Pill count (iii) Monitoring (iv) Referral of problem cases	(i) 1-week theoretical, (ii) 2-months practice, on the job training	Yes	(i) Mobile device (ii) Mobile phone	208 patients stable and at least 3 months on ART were randomly assigned to: community-based care (*N* = 96) and clinic-based care (*N* = 112): (i) 50% reduction clinical visits when in community-based care (ii) 5% LFU rate in both arms at 12 months follow-up time	Cluster RCT

Mozambique [22]	*PLWHA organized in community ART groups* in which 6 members are responsible for (i) Monthly drug refills (ii) Pill count (iii) Self-reporting (iv) Self-referral	(i) Information session on day of inclusion in a CAG (ii) 6 monthly interactive group sessions (iii) No formal training	No	No	1,301 patients at least 6 months stable on ART were enrolled CAG. After median follow-up time of 12,9 months: (i) 0.2% LFU (ii) 2.3% died (iii) 97.5% retained	Cohort study

RCT: randomised controlled trial; PLWHA: people living with HIV/AIDS; LFU: lost to followup; CAG: community ART groups.

**Table 2:** Application of conceptual framework to the programs in Kenya and Mozambique.

	**Essential elements to become expert patients**		
Country	Information and skills	Motivation to take responsibility	Peer support
Kenya [20, 21]	CCC (i) Trained in medical and psychosocial tasks (ii) Offer psychosocial support (iii) Deliver ART in the community (iv) Refer problem cases when needed	(i) Formal training (ii) Remuneration (iii) Strict control and supervision (iv) Proximity with fellow peers (v) Recognition by PLWHA and HCW	CCC (i) Better understanding of psychosocial barriers (ii) Rely on day-to-day experience living with HIV/AIDS (iii) Considered as advocates (iv) ↑ Communication between HCW and community

Mozambique [22]	Information loop between HCW and CAG members and sharing information and skills in the community: (i) ↑ Information circulating in the community (ii)↑ Skills of CAG members to (a) Provide ART in the community (b) Overcome daily obstacles to adherence (c) Offer psychosocial support (d) Refer problem cases when needed	(i) Direct social, health and economic benefits (ii) Proximity with fellow peers, reinforced during continuous contacts in the community and periodic group sessions (iii) Recognition by PLWHA and HCW	CAG members (i) Social relationship networks contributes to: (a) Continuous information sharing (b) Problem solving skills (ii) Proximity—mutual psychosocial or financial support to address common challenges

ART: antiretroviral treatment, CAG: community ART groups, CCC: community care coordinators, HCW: health care worker, PLWHA: people living with HIV/AIDS.

## References

[1] World Health Organization (WHO) Global HIV/AIDS response: epidemic update and health sector progress towards universal access. http://whqlibdoc.who.int/publications/2011/9789241502986_eng.pdf.

[2] Fox MP, Rosen S (2010). Patient retention in antiretroviral therapy programs up to three years on treatment in sub-Saharan Africa, 2007–2009: systematic review. *Tropical Medicine and International Health*.

[3] Kagee A, Remien RH, Berkman A, Hoffman S, Campos L, Swartz L (2011). Structural barriers to ART adherence in Southern Africa: challenges and potential ways forward. *Global Public Health*.

[4] Mills EJ, Nachega JB, Bangsberg DR (2006). Adherence to HAART: a systematic review of developed and developing nation patient-reported barriers and facilitators. *PLoS Medicine*.

[5] Wouters E, Van Damme W, Van Loon F, van Rensburg D, Meulemans H (2009). Public-sector ART in the Free State Province, South Africa: community support as an important determinant of outcome. *Social Science and Medicine*.

[6] Gifford AL, Groessl EJ (2002). Chronic disease self-management and adherence to HIV medications. *Journal of Acquired Immune Deficiency Syndromes*.

[7] Rueda S, Park-Wyllie LY, Bayoumi AM (2006). Patient support and education for promoting adherence to highly active antiretroviral therapy for HIV/AIDS. *Cochrane Database of Systematic Reviews*.

[8] Kober K, Van Damme W Expert patients and AIDS care: a literature review on expert programmes in high-income countries and an exploration of their relevance for HIV/AIDS care in low-income countries with severe human resource shortages. Institute of Tropical Medicine, Antwerp. http://www.hrhresourcecenter.org/node/389.

[9] Van Damme W, Kober K, Kegels G (2008). Scaling-up antiretroviral treatment in Southern African countries with human resource shortage: how will health systems adapt?. *Social Science and Medicine*.

[10] Torpey KE, Kabaso ME, Mutale LN (2008). Adherence suport workers: a way to address human resource constraints in antiretroviral treatment programs in the public health setting in Zambia. *PLoS One*.

[11] Stubbs BA, Micek MA, Pfeiffer JT, Montoya P, Gloyd S (2009). Treatment partners and adherence to HAART in Central Mozambique. *AIDS Care*.

[12] World Health Organization Task shifting: rational redistribution of tasks among health work- force teams: global recommendations and guidelines. http://www.who.int/healthsystems/TTR-TaskShifting.pdf.

[13] Bodenheimer T, Lorig K, Holman H, Grumbach K (2002). Patient self-management of chronic disease in primary care. *Journal of the American Medical Association*.

[14] Merten S, Kenter E, McKenzie O, Musheke M, Ntalasha H, Martin-Hilber A (2010). Patient-reported barriers and drivers of adherence to antiretrovirals in sub-Saharan Africa: a meta-ethnography. *Tropical Medicine and International Health*.

[15] Weidle PJ, Wamai N, Solberg P (2006). Adherence to antiretroviral therapy in a home-based AIDS care programme in rural Uganda. *Lancet*.

[16] Jaffar S, Amuron B, Foster S (2009). Rates of virological failure in patients treated in a home-based versus a facility-based HIV-care model in Jinja, southeast Uganda: a cluster-randomised equivalence trial. *The Lancet*.

[17] Mermin J, Were W, Ekwaru JP (2008). Mortality in HIV-infected Ugandan adults receiving antiretroviral treatment and survival of their HIV-uninfected children: a prospective cohort study. *The Lancet*.

[18] Kipp W, Konde-Lule J, Saunders LD (2010). Results of a community-based antiretroviral treatment program for HIV-1 infection in western Uganda. *Current HIV Research*.

[19] Pearson CR, Micek MA, Simoni JM (2007). Randomized control trial of peer-delivered, modified directly observed therapy for HAART in Mozambique. *Journal of Acquired Immune Deficiency Syndromes*.

[20] Wools-Kaloustian KK, Sidle JE, Selke HM (2009). A model for extending antiretroviral care beyond the rural health centre. *Journal of the International AIDS Society*.

[21] Selke HM, Kimaiyo S, Sidle JE (2010). Task-shifting of antiretroviral delivery from health care workers to persons living with HIV/AIDS: clinical outcomes of a community-based program in Kenya. *Journal of Acquired Immune Deficiency Syndromes*.

[22] Decroo T, Telfer B, Biot M (2011). Distribution of antiretroviral treatment through self-forming groups of patients in Tete province, Mozambique. *JAIDS Journal of Acquired Immune Deficiency Syndromes*.

[23] Van Olmen J, Ku GM, Bermejo R, Kegels G, Hermann K, Van Damme W (2011). The growing caseload of chronic life-long conditions calls for a move towards full self-management in low-income countries. *Globalization and Health*.

[24] Swendeman D, Ingram BL, Rotheram-Borus MJ (2009). Common elements in self-management of HIV and other chronic illnesses: an integrative framework. *AIDS Care - Psychological and Socio-Medical Aspects of AIDS/HIV*.

[25] Rifkin SB (2009). Lessons from community participation in health programmes: a review of the post Alma-Ata experience. *International Health*.

[26] Ware NC, Idoko J, Kaaya S (2009). Explaining adherence success in sub-Saharan Africa: an ethnographic study. *PLoS Medicine*.

[27] Callaghan M, Ford N, Schneider H (2010). A systematic review of task- shifting for HIV treatment and care in Africa. *Human Resources for Health*.

[28] Fulton BD, Scheffler RM, Sparkes SP, Auh EY, Vujicic M, Soucat A (2011). Health workforce skill mix and task shifting in low income countries: a review of recent evidence. *Human Resources for Health*.

[29] Pearson CR, Micek MA, Pfeiffer J (2009). One year after ART initiation: psychosocial factors associated with stigma among HIV-positive mozambicans. *AIDS and Behavior*.

[30] Philips M, Zachariah R, Venis S (2008). Task shifting for antiretroviral treatment delivery in sub-Saharan Africa: not a panacea. *The Lancet*.

[31] Rasschaert F, Pirard M, Philips MP (2011). Positive spill-over effects of ART scale up on wider health systems development: evidence from Ethiopia and Malawi. *Journal of the International AIDS Society*.

[32] Pascoe SJS, Langhaug LF, Mudzori J, Burke E, Hayes R, Cowan FM (2009). Field evaluation of diagnostic accuracy of an oral fluid rapid test for HIV, tested at point-of-service sites in rural Zimbabwe. *AIDS Patient Care and STDs*.

[33] Harries AD, Zachariah R, Lawn SD, Rosen S (2010). Strategies to improve patient retention on antiretroviral therapy in sub-Saharan Africa. *Tropical Medicine and International Health*.

[34] Lester RT, Ritvo P, Mills EJ (2010). Effects of a mobile phone short message service on antiretroviral treatment adherence in Kenya (WelTel Kenya1): a randomised trial. *The Lancet*.

[35] Garnett GP, Baggaley RF (2009). Treating our way out of the HIV pandemic: could we, would we, should we?. *The Lancet*.

